# Ocean Acidification and Increased Temperature Have Both Positive and Negative Effects on Early Ontogenetic Traits of a Rocky Shore Keystone Predator Species

**DOI:** 10.1371/journal.pone.0151920

**Published:** 2016-03-30

**Authors:** Patricio H. Manríquez, María Elisa Jara, Mylene E. Seguel, Rodrigo Torres, Emilio Alarcon, Matthew R. Lee

**Affiliations:** 1 Laboratorio de Ecología y Conducta de la Ontogenia Temprana (LECOT), Centro de Estudios Avanzados en Zonas Áridas (CEAZA), Coquimbo, Chile; 2 Centro de Investigación en Ecosistemas de la Patagonia (CIEP), Coyhaique, Chile; 3 Centro de Investigación: Dinámica de Ecosistemas marinos de Altas Latitudes (IDEAL), Punta Arenas, Chile; 4 Centro i~mar, Universidad de Los Lagos, Puerto Montt, Chile; University of Connecticut, UNITED STATES

## Abstract

The combined effect of ocean acidification and warming is expected to have significant effects on several traits of marine organisms. The gastropod *Concholepas concholepas* is a rocky shore keystone predator characteristic of the south-eastern Pacific coast of South America and an important natural resource exploited by small-scale artisanal fishermen along the coast of Chile and Peru. In this study, we used small juveniles of *C*. *concholepas* collected from the rocky intertidal habitats of southern Chile (39°S) to evaluate under laboratory conditions the potential consequences of projected near-future levels of ocean acidification and warming for important early ontogenetic traits. The individuals were exposed long-term (5.8 months) to contrasting *p*CO_2_ (ca. 500 and 1400 μatm) and temperature (15 and 19°C) levels. After this period we compared body growth traits, dislodgement resistance, predator-escape response, self-righting and metabolic rates. With respect to these traits there was no evidence of a synergistic interaction between *p*CO_2_ and temperature. Shell growth was negatively affected by high *p*CO_2_ levels only at 15°C. High *p*CO_2_ levels also had a negative effect on the predator-escape response. Conversely, dislodgement resistance and self-righting were positively affected by high *p*CO_2_ levels at both temperatures. High tenacity and fast self-righting would reduce predation risk in nature and might compensate for the negative effects of high *p*CO_2_ levels on other important defensive traits such as shell size and escape behaviour. We conclude that climate change might produce in *C*. *concholepas* positive and negative effects in physiology and behaviour. In fact, some of the behavioural responses might be a consequence of physiological effects, such as changes in chemosensory capacity (e.g. predator-escape response) or secretion of adhesive mucous (e.g. dislodgement resistance). Moreover, we conclude that positive behavioural responses may assist in the adaptation to negative physiological impacts, and that this may also be the case for other benthic organisms.

## Introduction

It is well understood that organisms in natural systems are continuously exposed to multiple stressors simultaneously [1,2]. Studies of the individual and synergistic effects of ocean acidification (OA) and warming on marine invertebrates have described effects on a wide variety of metabolic, morphological, and behavioural traits [3–9]. When these effects are negative and occur during the early ontogeny of an organism the result maybe an increased mortality for the species. Gastropod molluscs are major producers of carbonates [10], provide important ecosystem services [11] and support fisheries of high economic value [12]. The evidence suggests that multiple stressors can exacerbate negative effects when acting in concert or could be less severe than when acting individually. The analysis of independent factorial experimental studies of several stressor pairs reveals that for the combination of warming and OA a similar proportion of synergistic, antagonistic and additive interaction effects are found [13]. The same meta-analysis [13], considering all the possible pair-wise combinations of stressors, concluded that the cumulative effect of multiple stressors will often be worse than expected based on single stressor impacts. A more recent review on the multi-stressor impact of warming and OA on the life histories of marine invertebrates [14] indicated that additive negative effects were the most common. These were followed by antagonistic effects, in which high temperatures ameliorate some of the negative effects of OA [14]. Finally, the least common were the synergistic effects in which the negative effects of one stressor were exacerbated by another [14]. In summary, evidence highlights that OA and warming occur together and the combined effect of both is complex and not only the sum of their individual effects. Therefore, the combined effect of OA and warming must be investigated to better understand how they interact to affect individual organisms.

Warming and OA may induce changes in important behavioural, morphological and physiological traits of molluscs [15–19]. When these effects are negative and occur during the early ontogeny of an organism the result maybe an increased mortality for the species. The muricid gastropod *Concholepas concholepas* (Bruguière 1789) is a rocky shore keystone predator characteristic of the Pacific coast of South America [20]. Rocky shores where this keystone predator is absent have a radically different structure, in terms of the diversity and distributions of other intertidal organisms, compared to those where it is present [20]. Furthermore, *C*. *concholepas* supports an economically important fishery that provides significant income for many coastal communities. The management of this fishery, through the Management and Exploitation Areas for Benthic Resources scheme, by local communities has also led to increased protection of the coastal benthic ecosystem as a whole [21]. Previous studies of behavioural traits and the morphological development of *C*. *concholepas* under near-future scenarios of OA have revealed negative impacts on the avoidance response of juveniles when exposed to predators [15,22]. However, one of those studies did not detect changes in growth rates as a result of OA [15]. Physiological studies on small individuals of *C*. *concholepas* have observed that, in the early ontogeny of this species, short-term exposure to elevated levels of *p*CO_2_ increased standard metabolic rates and the expression of HSP70-like genes [23]. In marine calcifying organisms the consequences of OA on an important trait such as calcification might be also modified by warming [3,4,24]. Moreover, the increase in seawater temperature associated with global climate change is expected to impact all physiological processes, and many ecological interactions [25]. This suggests that an increase in either *p*CO_2_ or temperature alone, or the two stressors in combination might have an effect on metabolism; and this in turn may result in changes in morphological and behavioural traits during the early benthic ontogeny of this species. During the early benthic ontogeny *C*. *concholepas* can be found in the rocky intertidal habitats [26,27] where it naturally experiences daily fluctuations in pH [28] and temperature [29] and has thus evolved mechanisms to cope with these environmental stressors [29]. However, other studies have shown that some intertidal invertebrates are living near their physiological tolerance limits [30]. Therefore, near future conditions of OA and warming might push them to extreme suboptimal conditions that make them ideal model organisms for studying the impact of these stressors, either in isolation or in combination.

We manipulated water temperature and *p*CO_2_ levels to determine their effects on small juveniles of the keystone species *C*. *concholepas*. Small juveniles of *C*. *concholepas* have previously shown negative responses to OA alone [15,22]. However, in the intertidal environment they are exposed to natural variations in both temperature and pH that may exacerbate their responses to near-future scenarios combining elevated *p*CO_2_ and temperature. Thus, we hypothesised that negative changes in the physiological, morphological and behavioural responses of small juveniles of *C*. *concholepas* will be observed when they are exposed to the combination of elevated *p*CO_2_ and temperature.

## Materials and Methods

### Collection and rearing of juveniles

#### Ethics Statement

The model species used in the present study is not an endangered species (IUCN Red Data Books) and is not subject to restrictions under Chilean legislation; therefore specific permission for their collection was not required. Moreover, the individuals were collected from an open access shore and therefore no special authorization from a land or shore owner was required. All surviving individuals were returned to the same place that they were collected from at the end of the study.

Individuals of *Concholepas concholepas* (n = 50) with sizes ranging from 1.4 to 2.0 cm in size were collected from rocky intertidal platforms in Valdivia (southern Chile; 39°45'51''S; 73°23'54''W). The individuals were then transported to Coquimbo (northern-central Chile; 29°57'58''S; 71°21'17''W) where all the experiments were conducted. In the laboratory, individuals were marked with small numbered plastic discs to allow them to be identified throughout the course of the experiments.

**Fig 1:**
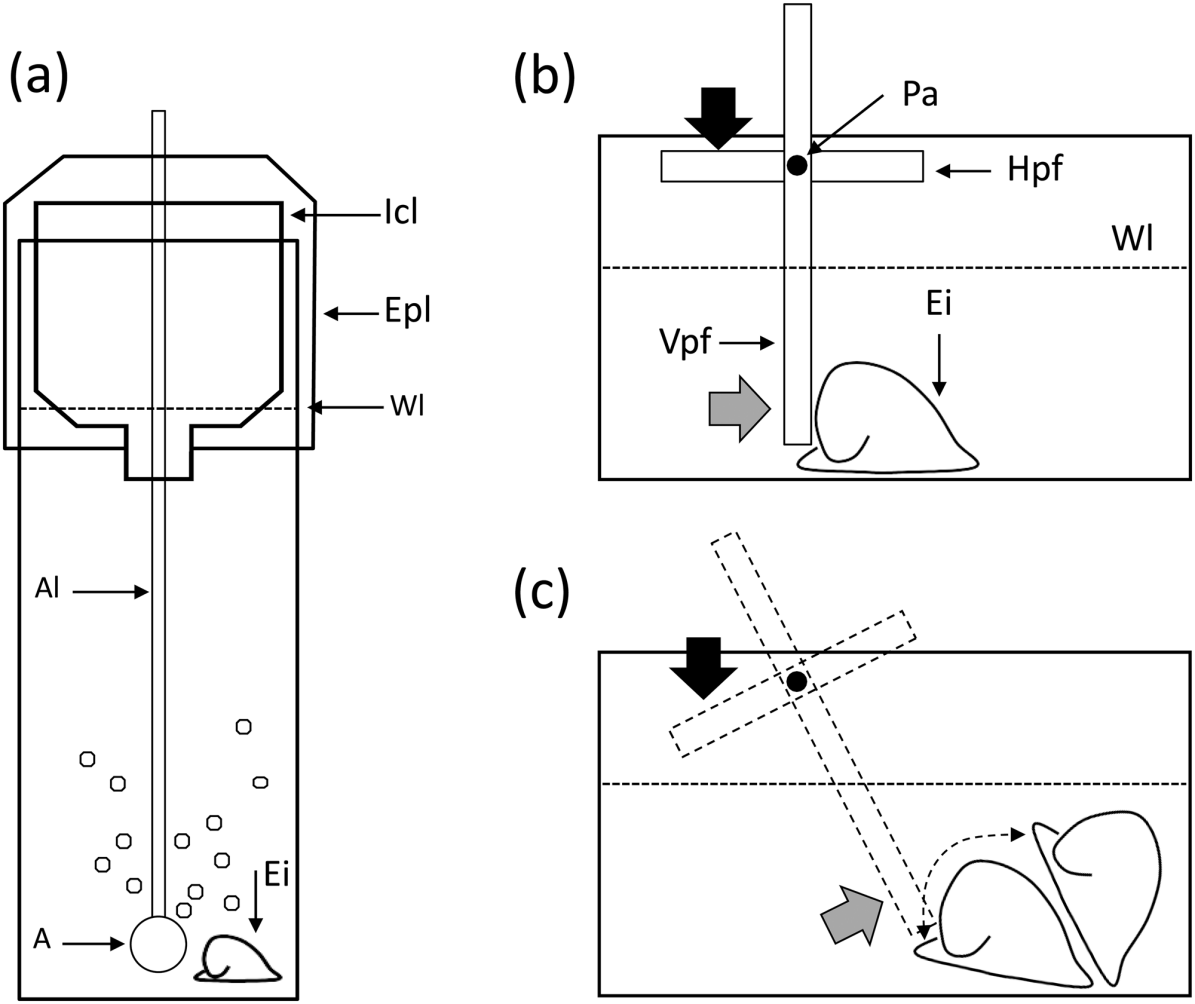
Schematic representations of the rearing bottles (a) and chamber used to measure dislodgement force (b-c). A: air stone, Al: plastic airline, Epl: external plastic lid; Icl: Internal conic lid; Pa: pivoting axis or fulcrum; Hpf: horizontal pivoting flap; Vpf: vertical pivotal flap; Wl: water level; Ei: experimental individual. In (c) the dashed contour depicts the position of the pivoting flap after the dislodgement force had been applied and the Ei was dislodged from the substratum. The black arrow depicts the point where the vertical force with the digital push-dynamometer was applied to the Hpf, and the grey arrow depicts the place where the resulting horizontal force of the Vpf was applied against the Ei.

The *C*. *concholepas* individuals were subjected to two phases in the laboratory, first an acclimatization phase followed by a treatment phase. To allow the individuals to adjust to laboratory conditions, during the first rearing phase (acclimatisation phase) the individuals were reared in a 7.5 L aquarium semi-immersed in a water bath to maintain the temperature at 15 ± 0.5°C. During this acclimatisation phase (ca. 2.1 months) the individuals were maintained with running seawater and fed *ad libitum* with fresh individuals of the mussel *Semimytilus algosus*. During the treatment phase (5.8 months) the *C*. *concholepas* were maintained in individual rearing chambers. Each chamber (Fig 1) was constructed from a 1.5 L plastic drinks bottle. The top third of the bottle was cut away and then inverted and placed into the bottom two thirds creating a chamber with an inverted cone at the top, this inverted cone proved effective in preventing the test organism from escaping. A plastic lid was then placed over the top of the rearing chamber. This second lid was pierced by an air tube which then passed through the cone to a diffuser in the lower portion of the rearing chamber.

For the treatment phase, individuals (n = 40) were assigned at random to rearing chambers, one individual per chamber. Rearing chambers were then assigned at random to one of four treatments combining temperature and *p*CO_2_ levels: 15°C / 500 μatm *p*CO_2_, 15°C / 1400 μatm *p*CO_2_, 19°C / 500 μatm *p*CO_2_ and 19°C / 1400 μatm *p*CO_2_. All treatments were made using 1 μm filtered seawater (hereafter FSW). Treatment levels were nominal and later confirmed by analysis of the seawater chemistry and temperature records (Table 1). The two selected *p*CO_2_ levels were chosen to match current-day levels in intertidal water in Herradura Bay, Coquimbo, Central Chile (ca. 500 μatm *p*CO_2_, Table 1) and over the actual maximum *p*CO_2_ levels records for the mid-depth waters that feed the upwelling off Coquimbo, Central Chile [31] which is close to the expected *p*CO_2_ average levels for the year 2150 under scenario RCP8.5/ECP8.5 [32]. The temperatures of 15°C and 19°C (± 0.5°C) were chosen to match average high temperatures near the collection site during the austral summer months and 4°C above this average, approximately the temperature increase predicted for the beginning of the next century [33]. During the treatment phase the chambers were semi-immersed in 2 large water tables (400 L each) with recirculating seawater maintained at the treatment temperatures by thermostatically controlled heat exchangers. The seawater contained in the rearing chambers was changed every second day with fresh conditioned 1 μm FSW produced in the acidification unit (see below). At the same time, small fresh *Semimytilus algosus* were added to each bottle and dead mussels removed to maintain a constant and sufficient food source of three live mussels. During the treatment period the chambers were cleaned weekly.

**Table 1:** Average (±SE) conditions of the seawater used to maintain small juveniles of *Concholepas concholepas* during the acclimatization and treatment phases. The higher *p*CO_2_ is based on rate of change in pH predicted by the most extreme scenario (RCP8.5 scenario) of atmospheric CO_2_ for the beginning of the next century. See Meinshausen et al. [34] for further details.

Level of *p*CO_2_ / Temperature °C	Temperature (°C)	pH at 25°C (pH units)	pH in situ (pH units)	AT (μmol kg^−1^)	*p*CO_2_ in situ (μatm)	[CO3^2–^] in situ (μmol kg^−1^SW)	Salinity	Ω calcite	Ω aragonite
**Live individuals**									
**Acclimatization phase**									
Seawater ^a^	14.67 (0.25)	7.79 (0.04)	7.95 (0.04)	2263.10 (6.22)	523.41 (58.04)	122.98 (9.03)	34.06 (1.14)	2.95 (0.21)	1.88 (0.13)
Current-day *p*CO_2_ / 15°C	14.85 (0.19)	7.79 (0.02)	7.93 (0.03)	2283.14 (4.28)	506.68 (16.90)	124.43 (4.74)	34.24 (0.07)	2.98 (0.11)	1.91 (0.07)
**Treatment phase**									
Seawater ^a^	16.92 (0.23)	7.84 (0.02)	7.96 (0.02)	2300.07 (3.35)	524.23 (22.30)	143.57 (4.98)	34.33 (0.02)	3.44 (0.12)	2.22 (0.08)
Current-day *p*CO_2_ / 15°C	15.72 (0.12)	7.71 (0.02)	7.85 (0.02)	1865.37 (52.31)	539.45 (9.74)	91.66 (4.74)	34.53 (0.03)	2.19 (0.11)	1.41 (0.07)
High *p*CO_2_ / 15°C	15.62 (0.09)	7.35 (0.01)	7.47 (0.01)	1942.44 (42.65)	1462.74 (25.37)	42.39 (1.86)	34.61 (0.04)	1.01 (0.04)	0.65 (0.03)
Current-day *p*CO_2_ / 19°C	19.15 (0.10)	7.65 (0.03)	7.73 (0.03)	1692.22 (69.71)	651.33 (25.54)	77.67 (6.06)	34.55 (0.03)	1.86 (0.15)	1.21 (0.09)
High *p*CO_2_ / 19°C	19.27 (0.05)	7.40 (0.01)	7.47 (0.02)	1906.12 (42.35)	1445.91 (33.21)	47.99 (2.09)	34.61 (0.10)	1.15 (0.05)	0.75 (0.03)
**Naked shells**									
Seawater ^a^	17.02 (0.22)	7.82 (0.04)	7.94 (0.03)	2298.90 (5.80)	557.57 (53.51)	137.67 (9.47)	34.45 (0.02)	3.30 (0.23)	2.13 (0.15)
Current-day *p*CO_2_ / 15°C	15.58 (0.07)	7.71 (0.03)	7.84 (0.03)	1812.66 (102.99)	532.11 (8.92)	86.30 (8.15)	34.64 (0.03)	2.06 (0.20)	1.33 (0.13)
High *p*CO_2_ / 15°C	15.56 (0.06)	7.34 (0.02)	7.46 (0.03)	1925.67 (88.69)	1478.54 (21.90)	40.76 (3.41)	34.69 (0.05)	0.97 (0.08)	0.63 (0.05)
Current-day *p*CO_2_ / 19°C	18.80 (0.27)	7.60 (0.05)	7.68 (0.06)	1623.67 (160.04)	647.85 (19.29)	65.14 (10.93)	34.65 (0.05)	1.56 (0.26)	1.01 (0.17)
High *p*CO_2_ / 19°C	19.12 (0.08)	7.40 (0.04)	7.47 (0.04)	1836.63 (108.66)	1375.24 (37.75)	47.16 (5.15)	35.09 (0.38)	1.13 (0.12)	0.73 (0.08)

^a^Seawater parameters were measured in the coastal seawater at Herradura Bay and correspond to the seawater used to generate the experimental conditions in the mesocosms during the acclimatization and treatment phase, and during the measurements of the potential corrosive effect of the experimental conditions on naked shells.

### Seawater acidification unit and carbonate system determination in the equilibrated seawater

Conditioned FSW with the two different *p*CO_2_ levels for the treatment chambers was generated in 4 polyethylene (230 L) reservoir tanks (see [34]). Each chamber was filled with conditioned FSW, and a continuous stream of either air (ca. 400 μatm CO_2_) or enriched CO_2_ air (ca.1200 μatm CO_2_) was bubbled through the water. Enriched CO_2_ air was produced by blending air and pure CO_2_ using mass flow controllers (see a full description of this type of seawater *p*CO_2_ control system in Torres et al. 2013 [34]. During the first rearing phase the seawater parameters (i.e., pH, temperature, salinity and total alkalinity) were measured twice a week in the seawater used to replace the water in the rearing aquarium (Table 1). However, in the treatment phase the parameters were measured twice a week in water samples taken from the rearing chambers before the water change took place. Therefore, these measurements represent the values achieved with the continuous stream of either air or enriched CO_2_ air plus the cumulative effect on the carbonate system of the 24 h of respiration, calcification and ammonium excretion associated with the metabolism of both trial snails and the prey in the 1.5 L chambers. The pH measurements were made in a closed 25 ml cell, thermostatically controlled at 25.0°C, with a Metrohm 713 pH meter (input resistance >10^13^ Ohm, 0.1mV sensitivity and nominal resolution 0.001 pH units) and a glass fixed ground-joint diaphragm electrode with Pt1000 (Metrohm 6.0257.000, Aquatrode plus) calibrated with 8.089 Tris buffer at 25.0°C [35]; pH values are therefore reported on the total hydrogen ion scale [35]. Total Alkalinity (A_T_) was determined by potentiometric titration in an open cell, according to Haraldsson et al. [36]. The accuracy was controlled against a certified reference material supplied by Andrew Dickson (Scripps Institution of Oceanography). The correction factor was approximately 1.002, corresponding to a difference of < 5μmol kg^-1^. Each sample was analysed using 2 or 3 replicates. Temperature and salinity were measured using a CTD (Ocean Seven 305). The pH, A_T_ and hydrographic data were used to calculate the rest of the carbonate system parameters (*p*CO_2_, total alkalinity, pH in situ, *p*CO_2_ in situ and [CO_3_^-2^]) and the saturation state of Ω aragonite and calcite, using CO_2_SYS software [37] set with Mehrbach solubility constants [38] refitted by Dickson & Millero [39].

### Growth

To estimate the effects of OA and warming on growth; shell size (the maximum length at the peristomal margin of the shell aperture or peristomal length), live wet weight and calcification rate via change in buoyant weight were measured at the beginning and end of the acclimatisation phase (2.1 months), and then at the end of the treatment phase (5.8 months). The differences in these variables were then converted in to percentage change over the course of the experimental period (specific growth rate). To estimate the effects on shell thickness we measured thickness at the base of the labral tooth that in *C*. *concholepas* forms a projection on the very end of the outer shell lip. This shell projection was selected as it can be measured without the need to euthanize the experimental individual and because in *C*. *concholepas* it is easily distinguishable and representative of the shell thickness at the growing edge (PHM personal observations). To standardise for shell size we computed a thickness index by dividing labral tooth thickness by the body size. The potential corrosive effect of the experimental conditions was evaluated by exposing 12 empty shells of small juveniles (ca. 3.0 cm) to each of the treatments (3 shells per treatment). Shell weight as a function of *p*CO_2_ and temperature levels was measured at the beginning and end of the exposure period (1 month). Since different rearing methods were used in each phase, in terms of seawater conditioning, changes in size and weight during the acclimatisation phase are reported but not included in the statistical analyses.

### Behavioural measurements

After 3 months of exposure in the treatment phase 3 behavioural traits were measured: (1) predation avoidance (2) self-righting time; the time span from the moment that an individuals is placed upside down to when it returns to the normal upright position. Finally, after an additional 1 month of exposure (3) the minimum force (tenacity) required to dislodge the individuals from the substratum was measured.

### Self-righting and predation avoidance

After 3 month in the treatment phase the individuals were removed from the chambers and allowed to acclimatise for 2 h to the experimental chambers. The self-righting measurements were conducted in a plexiglass chamber (19 × 10 × 6 cm; length, width, height) divided in to two equal parts by plastic mesh and filled with 0.5 L of 1 μm FSW equilibrated to the same temperature and *p*CO_2_ levels used during the treatment phase [see [15]. To accelerate the self-righting process a predatory crab *Acanthocyclus hassleri* [15,22,40] was placed on one side of the chamber and the experimental individuals on the other. The crab was introduced to the chamber 20 min before the test individual and a small air stone was used to circulate the crab-odour cues throughout the chambers. The air stone was then removed and the test individual carefully placed in the middle of the other side of the chamber from the crab and immobilised inside a piece of PVC tubing. After 10 min of acclimatisation the pipe was removed and the test individual was placed upside down. Self-righting time was considered as the total time span from the moment that the individuals were placed upside down to their return to the normal upright position.

The predation avoidance behaviour was observed in the same chambers described above. The snails were removed from the treatment conditions, and placed in the experimental chamber adjacent to the dividing mesh and immobilised inside a piece of PVC tubing. After 20 min of acclimatisation in the chamber a crab was introduced into the other side of the chamber and the PVC tubing removed to allow the snail to move freely. The final position in the chamber of the *C*. *concholepas* after 15 min of observation was recorded, with those snails moving to the wall of the chamber furthest away from the crab being considered to have displayed predator avoidance behaviour.

### Dislodgement resistance

The measurements were conducted after 4 months of exposure in the treatment phase, in a specially designed plexiglass chamber. The chamber (Fig 1b) consisted of a rectangular box (16 × 8 × 7.6 cm; length, width, height), along with two plexiglass flaps joined at 90° to each other and suspended from the top of the chamber from the axis or fulcrum created by the intersection of the two flaps. The longer of the two flaps (11.8 cm) hangs vertically in the chamber and the shorter (5.2 cm) parallel to the base of the chamber. A vertical force applied to the horizontal flap results in the rotation of the vertical flap applying a horizontal force across the base of the chamber. The chambers were filled with 0.3 L of 1 μm FSW equilibrated to the same temperature and *p*CO_2_ levels used during the treatment phase. The test individual was positioned in the base of the chamber with the left-hand edge of the shell in contact with the vertical flap. Temporary plastic barriers were used to prevent the snail from moving away and the snail it was given 20 min to attach itself to the base of the chamber. After the 20 min period the barriers were removed and the dislodgement force applied to the shell. To measure the minimum force required to dislodge the test individuals a vertical force was carefully applied with a digital push-dynamometer (PCE FM50) on the left side of the horizontal flap (Fig 1b) generating a horizontal force at the base of the vertical flap, which pushes against the shell of the test individuals. The push-dynamometer is able to measure a maximum force of 49 N (ca. 7.5 k), and the force applied can be held constant throughout the trial. As a general rule the snails with a larger foot area require more force to dislodge [41], therefore dislodgement force was standardised by pedal surface area. This was measured by tracing the outline of each attached foot onto a piece of glass, the outline was then transferred to paper, and a digital planimeter was then used to calculate the area of the foot.

### Metabolism

The metabolic rate measurements were made using small juveniles of *C*. *concholepas* after 4.8 (first series, 6–7 individual per treatment) and 5.8 months (second series, 5 individuals per treatment) in the treatment phase. All metabolic rates were measured individually in respirometric chambers (100 ml Schott Duran^®^ glass bottles) after incubation in each *p*CO_2_/temperature treatment. In both series, the seawater used for metabolism measurements was 0.45 μm FSW fully O_2_ saturated. The first series used standardised *p*CO_2_ (current-day *p*CO_2_ levels) and temperature (15°C) levels during the oxygen measurements. Individuals were starved for 24 h in 1 μm FSW prior to the experimental measurements, sufficient time to ensure that the metabolic rate was not affected by the last meal [15]. The first series was used to determine the shock response of the rearing conditions on metabolic rate. For the second series, individuals were again starved for 24 h and oxygen consumption was measured at the same four temperature and *p*CO_2_ combinations used for the rearing treatments. The second series was used to determine the long-term metabolic consequences of acclimatization to temperature and *p*CO_2_. A fibre optic oxygen-meter (Fibox 3, PreSens, Germany) was used to measure oxygen consumption (mg O_2_ g^−1^ h^−1^), zero calibration was performed using a Na_2_O_3_S solution (0% saturation) and 100% was calibrated using air-bubbled seawater. The temperature was stabilised using a temperature-controlled circulation bath. Individual measurements lasted for at least 60 min per individual, with the first ten minutes of data being eliminated to reduce the effects of manipulation stress on the final analyses. Special care was taken to prevent the oxygen levels from dropping below 70% of saturation to minimize stress effects [42]. Background respiration was determined from control experiments using chambers with treatment seawater but no organism. Oxygen consumption rates and background respiration were calculated using the linear decrease in oxygen concentration measured over intervals of between 30 and 90 min.

### Statistical analyses

Two-way ANOVA were used to evaluate whether temperature and *p*CO_2_ and the combination of these factors had an effect on the studied variables [43]. Where statistically significant effects were identified the ANOVA was followed by a Tukey's a posteriori HSD test to identify the significance of differences between pairs of conditions [44]. Differences in the proportion of individuals displaying avoidance behaviour after rearing at current-day and elevate *p*CO_2_ levels for each rearing temperature were analysed with a χ^2^ test. All statistical analyses were conducted using R version 3.0.2 [45]. Tenacity index and metabolic rates data (standard conditions) were log transformed, and metabolic rates data (treatment conditions) were rank transformed [46], to meet the assumptions of normality.

## Results

### Survival

During the 2 months acclimatisation phase the survival rate was 97.5%. Ten individuals were exposed to each combination of *p*CO_2_ and temperature, except for the current *p*CO_2_/high temperature treatment where only 9 individuals were exposed, due to the loss of one test organism during the acclimatisation phase. During the treatment phase survival was high for all treatments, with the lowest survival rate of 88.9% being recorded in the current *p*CO_2_/high temperature treatment.

### Growth

**Fig 2:**
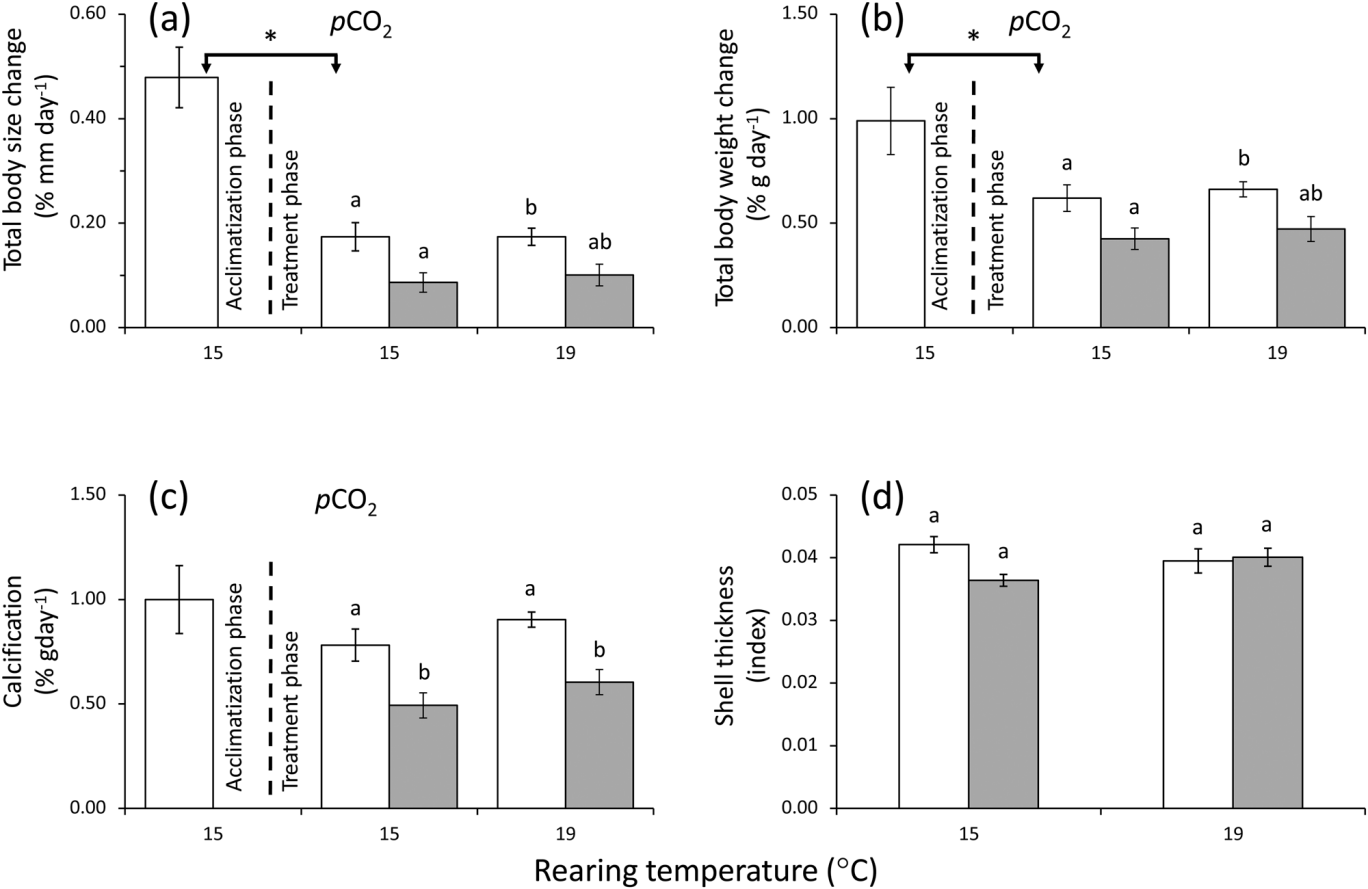
*Concholepas concholepas*. Effect of two different levels of *p*CO_2_ and temperature on the (mean ± SE) (a) percentage of total body size increase; (b) percentage of total body wet weight increase; (c) net calcification rate and (d) shell thickness index of small juvenile individuals reared for 5.8 months in the experimental conditions. For each panel, the designations ‘*p*CO_2_’ indicate significant *p*CO_2_ (two-way ANOVA). Open and filled bars represent measurements at current-day and high *p*CO_2_ levels respectively. Different letters above the bars indicate significant differences (p < 0.05) in Tukey's HSD *post hoc* test on the 2-way ANOVA analysis. The * above the open bars represent significant differences (p < 0.05) on the 1-way ANOVA analysis comparing the acclimatisation and the treatment phase at 15°C.

Average shell growth estimated from changes in body size was significantly higher during the acclimatisation phase than during the treatment phase at current-day *p*CO_2_ levels and at both temperatures (1-way ANOVA, *F*_2,27_ = 17.518, p < 0.05; Fig 2a). During the treatment phase *p*CO_2_ level did have a significant effect on shell growth (2-way ANOVA p < 0.05; Fig 2a; Table 2). At 15°C, elevated *p*CO_2_ levels caused significantly reduced growth as measured by changes in body size (Tukey's HSD test p < 0.05; Fig 2a). However, no significant differences were found at 19°C (Tukey's HSD test; p > 0.05; Fig 2a; Table 2). During the treatment phase there was no significant effect on shell growth due to temperature and *p*CO_2_ levels in combination, nor by temperature alone (Fig 2a; Table 2).

**Table 2:** *Concholepas concholepas*. Two-way ANOVAs investigating the effect of different combinations of temperature (15 and 19°C) and *p*CO_2_ (500 and 1400 μatm) levels on three body traits changes measured in small juveniles after 5.8 months of rearing in the experimental conditions (Fig 2a and 2d). Values in bold are significant at p < 0.05.

**Changes in body size**					
Source of variation	DF	SS	MS	*F*	p
Temperature	1	0.000	0.000	0.001	> 0.05
*p*CO_2_	1	0.059	0.059	14.080	**< 0.005**
Temperature × *p*CO_2_	1	0.001	0.001	0.106	> 0.05
Error	33	0.139	0.004		
**Changes in total weight**					
Source of variation	DF	SS	MS	*F*	p
Temperature	1	0.007	0.007	0.251	> 0.05
*p*CO_2_	1	0.342	0.342	11.646	**< 0.005**
Temperature × *p*CO_2_	1	0.000	0.000	0.001	> 0.05
Error	33	0.969	0.029		
**Changes in calcification**					
Source of variation	DF	SS	MS	*F*	p
Temperature	1	0.108	0.108	2.812	> 0.05
*p*CO_2_	1	0.887	0.887	23.047	**< 0.005**
Temperature × *p*CO_2_	1	0.001	0.001	0.030	> 0.05
Error	33	1.270	0.039		
**Changes in shell thickness**					
Source of variation	DF	SS	MS	*F*	p
Temperature	1	0.000	0.000	0.078	> 0.05
*p*CO_2_	1	0.000	0.000	2.902	> 0.05
Temperature × *p*CO_2_	1	0.000	0.000	4.038	> 0.05
Error	33				

Average growth estimated from changes in total wet weight was significantly higher during the acclimatisation phase than during the treatment phase at current-day *p*CO_2_ levels and at both temperatures (1-way ANOVA, *F*_2,26_ = 2.212; p < 0.05; Fig 2b). Again, during the treatment phase, *p*CO_2_ level did have a significant effect on total wet weight (2-way ANOVA p < 0.05; Fig 2b; Table 2; Fig 2b). However, only the *post hoc* comparison between current-day *p*CO_2_ at 15°C and future *p*CO_2_ at 19°C was significant (p < 0.05); the differences in total wet weight between the *p*CO_2_ levels within the two temperature treatments were not significant (Fig 2b). During the treatment phase there was no significant effect on growth due to temperature and *p*CO_2_ level in combination nor by temperature alone (Fig 2b; Table 2).

Shell calcification of live individuals as measured by changes in buoyant weight during the acclimatisation phase was not significantly different to that recorded during the treatment phase at current-day *p*CO_2_ levels and at both temperatures (1-way ANOVA, *F*_2,17_ = 1.207; p > 0.05; Fig 2c). However, during the treatment phase at both temperatures shell calcification was significantly lower under future *p*CO_2_ levels (Tukey's HSD test p < 0.05; Fig 2c). During this phase temperature and *p*CO_2_ in combination had no significant effect on total shell calcification (Fig 2c; see Table 2).

There was no significant effect of temperature, *p*CO_2_ levels nor the interaction between these two variables on the shell thickness index of live individuals (2-way ANOVA p > 0.05; Fig 2d; see Table 2). No significant differences were found in the dry weights of the empty shells at the beginning of the rearing period (1-way ANOVA; *F*_3,8_ = 0.04; p > 0.05; Table 3). However, after 1 month of exposure to the experimental treatments empty shell weight loss was significantly increased by temperature (2-way ANOVA; *F*_1,8_ = 6.014; p < 0.05), *p*CO_2_ (2-way ANOVA; *F*_1,8_ = 22.752; p < 0.05) and the interaction between *p*CO_2_ level and temperature (2-way ANOVA; *F*_1,8_ = 11.347; p < 0.05; Table 3). At both rearing temperatures more shell material was lost in those empty shells exposed to elevated *p*CO_2_ levels (1-way ANOVA, *F*_1,4_ = 18.516 p < 0.05; 15°C; 1-way ANOVA, *F*_1,4_ = 8.221; p < 0.05; Table 3).

**Table 3:** *Concholepas concholepas*. Initial dry weight (g) and effects of contrasting rearing conditions temperature (15 and 19°C) and *p*CO_2_ (500 and 1400 μatm) levels on the mean (± SE) temporal dry weight losses (mg · day^-1^) in naked shells after 1 month of rearing in the experimental conditions. The measurements were done on 4 shells at the beginning of the experiment and the after 1 month of rearing under the contrasting conditions of *p*CO_2_ and temperature. The initial weight of naked shell assigned to each experimental temperature and *p*CO_2_ conditions were compared by 1-way ANOVA. Similar superscripts indicate lack of significant differences between treatments (p > 0.05). Weight losses were compared by 2-way ANOVA.

Rearing phase	Initial weight (g)	weight loss (mg · day^-1^)
15°C / Current-day *p*CO_2_	5.3643 (1.8007)^a^	0.2622 (0.0939)*,^a^
15°C / high *p*CO_2_	5.8227 (2.0895)^a^	0.7622 (0.0723)*,^b^
19°C / Current-day *p*CO_2_	4.9989 (1.2197)^a^	0.3178 (0.0222)*,^a^
19°C / high *p*CO_2_	5.3218 (1.5483)^a^	0.4022 (0.0248)*,^b^

* = significantly affected by *p*CO_2_, temperature and by the interaction between temperature and *p*CO_2_ levels (2-way ANOVA; p < 0.05). Different superscripts indicate significant differences between mean values (Tukey's HSD test p < 0.05) within each experimental temperature. See text for details.

### Self-righting and predation avoidance

Irrespective of the temperature, predation avoidance behaviour was observed in all individuals exposed to current-day *p*CO_2_ levels. Individuals exposed to elevated *p*CO_2_ levels exhibited a reduced frequency of predation avoidance at 15°C (χ^2^_(1)_ = 10.77; p < 0.05) and 19°C (χ^2^_(1)_ = 4.56; p < 0.05) (Table 4). During the observation period (15 min) the individuals reared at elevated *p*CO_2_ levels tended to remain motionless or displayed short and random movements.

**Table 4:** *Concholepas concholepas*. Proportion of individuals displaying predator avoidance and self-righting behaviour. Number in parenthesis represents the sample size. The measurements were done after 3 month of rearing under contrasting conditions of *p*CO_2_ and temperature. Proportion of avoidance success between *p*CO_2_ levels within each rearing temperature were analyzed with a χ^2^ test. Different superscripts indicate significant differences between treatments (p < 0.05).

Rearing condition	Avoidance success	Self-righting success
15°C		
Current-day *p*CO_2_	1.0 (10)^a^	1.0 (10)
High *p*CO_2_	0.3 (9)^b^	1.0 (9)
19°C		
Current-day *p*CO_2_	1.0 (9)^a^	1.0 (9)
High *p*CO_2_	0.6 (10)^b^	1.0 (10)

**Fig 3:**
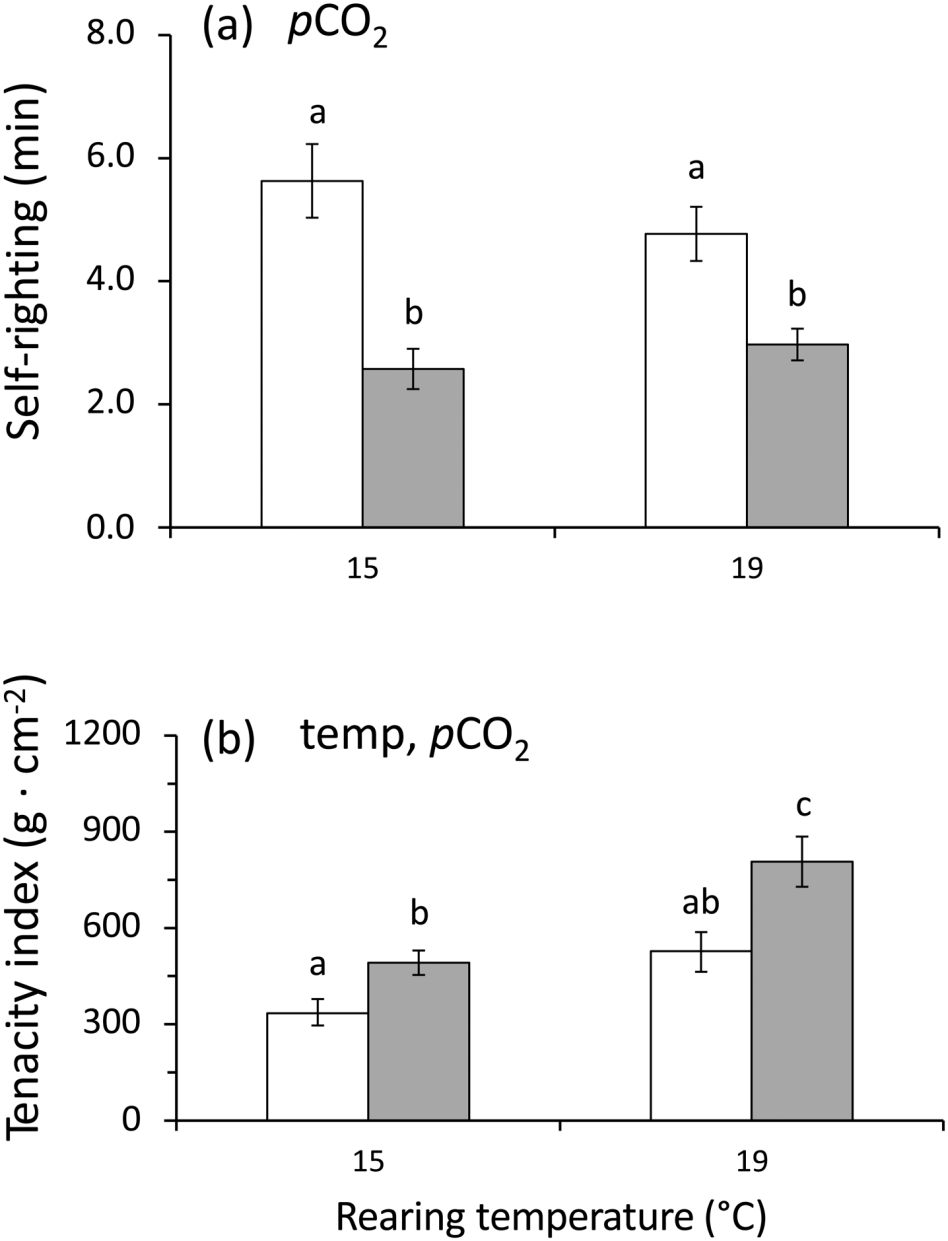
*Concholepas concholepas*. Effect of two different levels of *p*CO_2_ and temperature on the (mean ± SE) (a) self-righting time and (b) force required to dislodge small juvenile individuals reared for 4 months in the experimental conditions. For each panel, the designations ‘temp’ and ‘*p*CO_2_’ indicate significant temperature or *p*CO_2_ (2-way ANOVA). Open and filled bars represent measurements at current-day and high *p*CO_2_ levels respectively. Different letters above the bars indicate significant differences (p < 0.05) in Tukey's HSD *post hoc* test on the 2-way ANOVA analysis.

Self-righting was observed in individuals exposed to all of the experimental treatments (Table 4). There was no interaction between temperature and *p*CO_2_ for self-righting nor for temperature alone (Table 4). At elevated *p*CO_2_ levels self-righting times were significantly faster in individuals reared at high *p*CO_2_ levels at both 15°C (Tukey's HSD test; p < 0.05) and 19°C (Tukey's HSD test; p < 0.05; Fig 3a). Temperature alone and temperature and *p*CO_2_ in combination had no effect on self-righting times (Table 4).

### Dislodgement resistance

Between 190 and 1200 grams per cm^2^ of pedal area were required to dislodge the individuals (Fig 3b). At 15°C significantly more force was required to dislodge individuals at high *p*CO_2_ levels compared to low *p*CO_2_ levels, the same result was observed at 19°C. At high *p*CO_2_ levels, significantly more force was required to dislodge individuals at 19°C compared to 15°C. However, at low *p*CO_2_ levels the force required to dislodge individuals was not significantly different between temperatures. The combination of temperature and *p*CO_2_ had no significant effect on tenacity (Table 5).

**Table 5:** *Concholepas concholepas*. Two-way ANOVAs investigating the effect of different combinations of temperature (15 and 19°C) and *p*CO_2_ (500 and 1400 μatm) levels on two behavioural responses measured in small juveniles after 5.8 months of rearing in the experimental conditions (Fig 3a and 3b). Values in bold are significant at p < 0.05. Tenacity index data set was log transformed to meet assumptions of normality.

**Self-righting time**					
Source of variation	DF	SS	MS	*F*	p
Temperature	1	1.23	1.23	0.699	> 0.05
*p*CO_2_	1	55.72	55.72	31.537	**< 0.0001**
Temperature × *p*CO_2_	1	3.74	3.74	2.116	> 0.05
Error	34	60.07	1.77		
**Tenacity index**	DF	SS	MS	*F*	p
Source of variation					
Temperature	1	1.595	1.595	15.849	**< 0.0005**
*p*CO_2_	1	2.605	2.605	25.878	**< 0.0005**
Temperature × *p*CO_2_	1	0.090	0.090	0.892	> 0.05
Error	33	3.322	0.101		

### Metabolism

**Fig 4:**
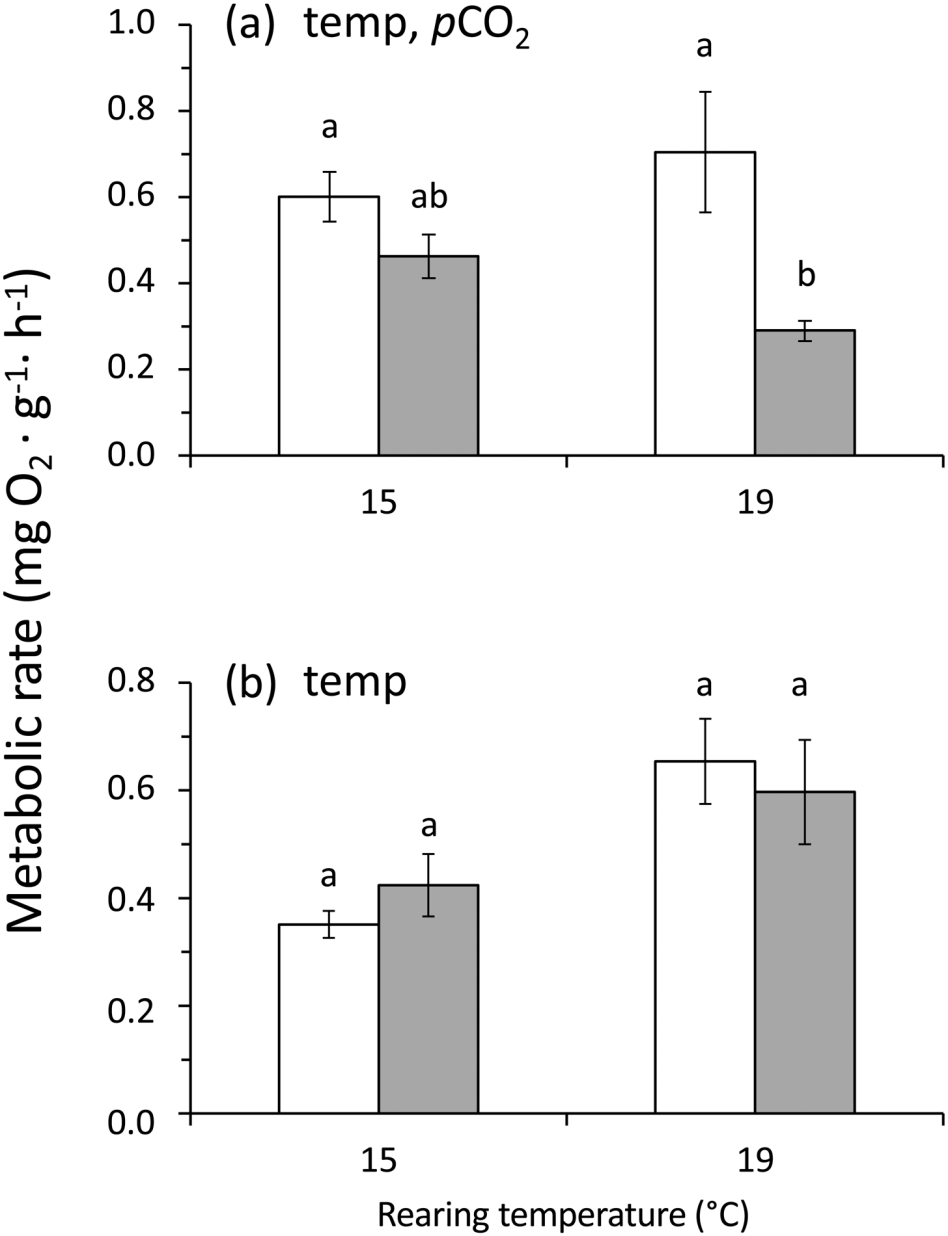
*Concholepas concholepas*. Effect of two different levels of *p*CO_2_ and temperature on the metabolic rate (mean ± SE) measured in small juvenile individuals after 4.8 (a) and 5.8 (b) months of rearing in the experimental conditions. For each panel, the designations ‘temp’ and ‘*p*CO_2_’ indicate significant temperature or *p*CO_2_ (2-way ANOVA). Open and filled bars represent measurements conducted in individuals reared at current-day and high *p*CO_2_ levels respectively. In (a) the metabolic rates were measured under standard conditions of *p*CO_2_ (current-day) and temperature (15°C). However, in (b) the metabolic rates were measured at the same conditions used during rearing. Different letters above the bars indicate significant differences (p < 0.05) in Tukey's HSD *post hoc* test on the 2-way ANOVA analysis.

The results of the first series of measurements indicate a reduced metabolic rate in those individuals reared under high *p*CO_2_ conditions at both temperatures. Temperature and *p*CO_2_ level individually had a significant effect on metabolic rate (2-way ANOVA; p > 0.05; Table 6). At both rearing temperatures the metabolic rate was significantly reduced at elevated *p*CO_2_ levels. (Tukey's HSD test; p < 0.05; Fig 4a). The combination of temperature and *p*CO_2_ level had no significant effect on metabolic rate (Table 6). As expected, given the thermodynamic principles for ectothermic organisms, the second series of metabolic rates were higher at the higher temperature (Fig 4b). Only temperature had a significant effect on the metabolic rate (2-way ANOVA; p < 0.05; Table 6). The *p*CO_2_ level alone and the combination of *p*CO_2_ and temperature had not a significant effect on the metabolic rate (2-way ANOVA; p > 0.05; Table 6).

**Table 6:** *Concholepas concholepas*. Two-way ANOVAs investigating the effect of different combinations of temperature (15 and 19°C) and *p*CO_2_ (500 and 1400 μatm) levels on the metabolic rate measured in small juveniles after 4.8 and 5.8 months of rearing in the experimental conditions (Fig 4a and 4b). Values in bold are significant at p < 0.05. Data for metabolic rate under standard conditions was log transformed, and data for metabolic rate under treatment conditions were rank transformed.

**Metabolic rate at standard conditions**					
Source of variation	DF	SS	MS	*F*	p
Temperature	1	0.761	0.761	5.319	**< 0.05**
*p*CO_2_	1	1.511	1.511	10.840	**< 0.005**
Temperature × *p*CO_2_	1	0.190	0.190	1.327	> 0.05
Error	23	3.291	0.143		
**Metabolic rate at rearing conditions**					
Source of variation	DF	SS	MS	*F*	p
Temperature	1	7.745	7.745	12.531	**< 0.005**
*p*CO_2_	1	0.122	0.122	0.205	> 0.05
Temperature × *p*CO_2_	1	0.087	0.087	0.873	> 0.05
Error	12	7.136	0.595		

## Discussion

In the present study we assessed the morphological, behavioural and physiological responses of small juveniles of the marine gastropod *Concholepas concholepas* following exposure to increased *p*CO_2_ and temperature over a period of 5.8 months without food limitation. We did not attempt to determine the precise physiological mechanisms underpinning the observed responses. However, our results showed that the effects of the two investigated stressors induce in *C*. *concholepas* behavioural modifications, increased tenacity and faster self-righting, that might assist in coping with the negative physiological impacts of reduced growth and chemosensory impairment, manifest in reduced predator avoidance behaviour. Whether or not the behavioural responses described in the present study are behavioural decisions or direct physiological effects that lead to a given response are still unknown. In fact, some of the behavioural responses described in the present study such as tenacity, self-righting and escape behaviour can also reflect direct physiological effects on the underlying mechanisms, such as changes in chemosensory capacity.

### Growth

The observed higher average body size change during the acclimatisation phase compared to the treatment phase at 15°C in the present study agrees with previous observations and presumably reflects the characteristic decrease in growth rate after the fast exponential growth normally observed during the early ontogeny of this species [47]. Total weight change, calcification and shell thickness were not significantly affected by temperature. This suggests that within the investigated thermal range (15 to 19°C) this species can cope with the temperature increase. This study has also demonstrated that irrespective of the treatment temperature calcification in *C*. *concholepas* was negatively affected by high *p*CO_2_ levels. However, the combination of high *p*CO_2_ and temperature had neither a synergistic nor antagonistic effect. Thus the observations of previous studies [48] in which the negative effects of elevated *p*CO_2_ levels were reduced by increases in temperature, were not observed in the present study. The presence of significantly reduced shell calcification at high *p*CO_2_ levels and the absence of a significant combined effect of *p*CO_2_ and temperature on shell calcification has also been reported in the mussel *Mytilus chilensis* [17]. The lack of interactive effects of *p*CO_2_ and temperature levels in *C*. *concholepas* could mean that the temperature range used in the present study was not sufficient to induce negative or synergistic effects.

Live shells of molluscs do not behave as empty shells do because many calcifying species are able to upregulate their metabolism to compensate for the change in conditions [49], thus the results of the exposures of the empty shells must be interpreted taking this in to account. The shell in juveniles of *C*. *concholepas* is comprised mainly of aragonite which makes them more vulnerable to dissolution [50]. This is supported by the observations of this study where the loss of empty shell material was higher under high *p*CO_2_ conditions. A previous study reported no significant differences in the loss of material from empty shells of *C*. *concholepas* maintained under acidified conditions (716 and 1036 μatm *p*CO_2_, [15]). Although in both this and the previous study the shells were exposed for 30 days to the experimental conditions, the previous study was conducted with shells ca. 2.5 cm in size and at an average temperature of 12 ± 1°C (15) whereas the present study used shells ca. 3.0 cm in size at temperatures of 15 ± 1°C and 19 ± 1°C (Table 1). At both the experimental temperatures used in the present study, more shell material was lost from empty shells maintained under high *p*CO_2_ levels (Table 3) and lower values of shell calcification in live individuals were detected under high *p*CO_2_ conditions (Fig 4). Although shells of this species have a periostracum it does not remain attached to the shell surface after death. In the present study this external and protective organic layer was not present at the beginning of the experiment with the empty shells. This suggests that shells deprived of a periostracum may be more vulnerable to shell corrosion than the shells of live individuals [24]. The fact that more surface of the shell is exposed to seawater when the shell is empty than when it contains a living individual should be also considered beyond the lack of perisotracum. Under high *p*CO_2_ conditions the seawater was saturated for calcite, in the case of live individuals, and close to saturation for empty shells, but it was not saturated for argonite in either case and thus the seawater was corrosive for the shells. This suggests that live individuals of *C*. *concholepas* at high *p*CO_2_ levels are more vulnerable to shell corrosion. This might in part explain the high vulnerability of *C*. *concholepas* shells in southern Chile to shell-boring invertebrates [51], as observed around the site were the test individuals were collected (39 °S) and in central northern Chile (29–32°S, Manríquez PH pers. obs.). This susceptibility could be higher in southern Chile due to the natural and higher variability in seawater pH in response to significantly higher freshwater inputs to the coastal system (e.g. [52]) and higher *p*CO_2_ sequestration (e.g. [53]). Thus the main structure associated with protection from predators and unfavourable conditions in *C*. *concholepas* and other shelled molluscs could be severely threatened under near future OA conditions.

Although in the present study we recorded the effects of elevated *p*CO_2_ levels on shell calcification (live individuals) and shell corrosion (empty shells) measured as change in buoyant weight, no significant effects on the shell thickness of live individuals were found. This suggests that the low calcification recorded in live individuals of *C*. *concholepas* at high *p*CO_2_ levels was not enough to produce detectable differences in shell thickness, that this part of the shell is not susceptible to changes in shell calcification or that those changes take places in other areas of the shell and not at the growing edge. We do not know why significant reductions in shell calcification did not take place along with reductions in shell thickness. Differences in the weight and morphology of shells of small *C*. *concholepas* collected from sites along the Chilean coast with different SST and carbonate system parameters together with an ontogenetic shift in shell mineralization of those individuals has been reported [50]. Likewise, increases in temperature at current day *p*CO_2_ levels negatively affected calcium content in the barnacle *Semibalanus balanoides* but not the growth rate [54]. Therefore, it is possible that the low calcification recorded in the present study was occurring along with changes in calcium carbonate mineralization that do not alter shell thickness in *C*. *concholepas*.

With respect to shell corrosion measured as change in buoyant weight it should be borne in mind that under natural conditions only the exterior surface of the shell is exposed to the environment and that the interior surface is in contact with body fluids, which have been demonstrated in the case of bivalves to be more corrosive than the surrounding seawater during low-tide periods [49]. This study [49] indicated that well-fed and healthy molluscs are able to maintain the inner nacreous layer of their shells, even under corrosive seawater conditions. This suggests that it is difficult to infer shell corrosion based on empty shells exposed to contrasting conditions of *p*CO_2_ alone or in combination with other stressors such as temperature. Therefore, measures based on empty shells exposed to different experimental conditions of *p*CO_2_ and temperature may overestimate true levels of shell corrosion.

### Behavioural measurements

The positive effect of high *p*CO_2_ levels alone on self-righting confirms the findings of a previous study on the same species [15]. This could be an important response in the early ontogeny for *C*. *concholepas*, or indeed any other species with a similar life history inhabiting the rocky intertidal habitats, where dislodgement is common and predators are abundant. The absence of predator-avoidance behaviour under high *p*CO_2_ levels also agrees with a previous study [22]. Likewise, the absence of avoidance behaviour, with individuals remaining motionless in the experimental chamber when reared under high *p*CO_2_ conditions, confirms the observations of a previous study in which *C*. *concholepas* were reared in the presence of the shell-crushing predatory crab *A*. *hassleri*, a visual predator [15,22]. This suggests that motionlessness and high tenacity might confer less vulnerability and may also be an energy saving strategy. As with other studies, the absence of avoidance behaviour may also suggest the presence of a negative effect of elevated *p*CO_2_ levels on the chemosensory detection of predators [54–57].

Gastropod molluscs depend on mucus for locomotion and for attaching themselves to the substratum [58]. However, in *C*. *concholepas*, as in other similar gastropods, foot suction also plays an important role in mechanical adhesion [59]. Individuals of *C*. *concholepas* routinely attach themselves to rocks with notable force. In fact, rocky intertidal shellfish gatherers and divers use a tool called a "chope", a short steel spike, to pry them from the substratum [60]. More force per unit of foot area is needed to dislodge *C*. *concholepas* reared at high *p*CO_2_ levels at both rearing temperatures, which suggests a positive effect of an increase in *p*CO_2_ levels on tenacity. This highlights that increased and sustained near future conditions of *p*CO_2_ and temperature could favour positive behavioural responses in small juveniles of *C*. *concholepas*; speedy self-righting and high tenacity. Both behavioural responses, self-righting and tenacity, involve mucus production, which in gastropods is described as a highly demanding activity in terms of energy use [61,62]. Thus, our results suggest that individuals of *C*. *concholepas* maintained under stressful conditions (high *p*CO_2_ levels) might reduce their metabolic activity and growth to meet the high energy demands associated with important behavioural responses such as self-righting and adhesion without reducing their metabolic activity. This capacity is of particular importance in intertidal environments where individuals are exposed during low tides to environmental hypoxia [63], and considerable variability in pH [28] and temperature [29]. Therefore, as has been suggested recently in the literature, we conclude that the effects of OA and warming on animal behaviour are significant and might play an important role in determining the persistence of species and their success in ecological communities [64].

### Metabolism

The average metabolic rates reported in the present study are within the range previously described for small juveniles of *C*. *concholepas* [15,23]. However, irrespective of the treatment temperature, when measurements were conducted under standard conditions (15°C and current-day *p*CO_2_ levels) the present study found the existence of a significant reduction in the metabolic rates of individuals reared at high temperature and *p*CO_2_ levels. This suggests the existence of a stress response in individuals moved from the treatment conditions to the measurement conditions at high temperatures. Metabolic depression in response to environmental stress has been recorded for virtually all the major animal phyla (see [65] for a review). In marine gastropods decreased metabolic rates have been linked with a strategy of matching demand to the availability of oxygen, and an increase in tissue concentrations of end-product metabolites indicating an increased reliance on anaerobic metabolism [66]. Individuals of *C*. *concholepas* inhabiting rocky intertidal habitats are commonly exposed to long periods (ca. 3 h) of air exposure and desiccation stress during low tides. In this environment invertebrates have evolved metabolic adaptations to environmental hypoxia and anoxia [63]. The metabolic depression in response to high *p*CO_2_ levels could be interpreted as an effort to conserve energy for future energy demanding activities in individuals inhabiting intertidal pools where they are exposed to a succession of pH fluctuations [67]. This agrees with previous studies that suggest that the primary metabolic mechanism for responding to OA involves energy budget reallocation [68–70]. On the other hand, when measurements were done at the treatment levels of temperature and *p*CO_2_ this reduction was not detectable. The metabolic rate was significantly affected by temperature, but not by *p*CO_2_ level nor by the interaction between both stressors. Marine organisms within a certain range of temperature have the capability of acclimating their metabolic rate [71,72]. This suggests that under global change scenarios no significant effects of OA on the metabolism of *C*. *concholepas* will occur. However, significant effects of ocean warming on the metabolism of this species are expected.

The present study found that temperature alone did not significantly affect most of the investigated traits. The only investigated traits that were significantly affected by temperature were tenacity and the metabolic rate. This suggests that the isolated effect of temperature is variable and depends on the response trait being measured. Moreover, the present study found a lack of interactive effects of *p*CO_2_ levels and temperature on all the investigated physiological, morphological and behavioural traits. This may be because the experimental temperature range used in the present study (i.e. 15–19°C) was not wide enough to include temperatures close to the upper thermal limit of *C*. *concholepas* and thus induce additive negative (sum of their individual effect of the stressors), synergistic negative (greater than the sum of their individual effects of the stressors) or antagonistic (contrasting actions of the stressors) effects of temperature and *p*CO_2_ that may occur when the individuals are under higher levels of stress [73]. The effects of temperature on several traits in marine invertebrates have been shown to depend on the thermal variability experienced by the species in the field [73,74]. Sea-surface temperature along the coast of Valdivia, near the rocky intertidal zone where the individuals were collected (39°S), ranges between 10 and 15°C with an average (± SD) of 12°C (± 1) (Manríquez PH unpublished data). Therefore the control temperature conditions used in the present study (15°C) represent a temperature near the maximum recorded for the collection site. At this site settlers and small juveniles of *C*. *concholepas* inhabiting rocky intertidal habitats may face higher temperatures during the short (ca. 3 h) exposure to air temperatures during low tide. As a consequence, the population used in the present study is rarely exposed to steady high temperatures, such as the 19°C used in this study. In consequence our results suggest that the combined effects of warming and acidification on the early benthic ontogeny of *C*. *concholepas* is complex (negative or positive depending on the analysed trait) and without significant interaction between both stressors. A potential explanation for the different effects of elevated *p*CO_2_ and temperature on some of the investigated traits could be that the underlying mechanism by which each individual stressor drives the investigated traits is different. Future studies are needed to go beyond this relatively moderate warming and describe the underlying mechanisms in order to fully understand the combined effect of warming and acidification on *C*. *concholepas*.

Despite the fact that intertidal organisms are presumably adapted to daily fluctuations in *p*CO_2_ and temperature levels we conclude that sustained exposure to the expected increase in *p*CO_2_ might have negative consequences for some important traits in the early life-history of this keystone species inhabiting the rocky intertidal zone. This is reinforced by the metabolic depression found in individuals moved from rearing conditions at high temperature and *p*CO_2_ levels to standard conditions similar to current day conditions. This might be to the particular importance of acute changes in temperature and pH that organism in intertidal rock pools face [67]. We also conclude that in the early ontogeny of *C*. *concholepas* the near future increase in *p*CO_2_ levels will not affect their metabolic rate but will have sub-lethal and negative consequences on shell growth and integrity (e.g. calcification and corrosion). However, the negative consequences of elevated *p*CO_2_ levels on predator avoidance behaviour in this keystone species might have lethal consequences with knock on effects on population dynamics and community structure. Though it needs to be established whether the negative effect of reduced avoidance behaviour and possible weaker shells are compensated for by the increased dislodgement force and self-righting times. In agreement with a recent review [64] we conclude that without considering the effects of OA and warming on animal behaviour we will have a limited capacity to predict the ecological effects of ocean change.

Our results suggests that the consequences of elevated temperatures and *p*CO_2_ level on a southern population of *C*. *concholepas* will be less severe than expected because of the absence of negative effects of increased temperature alone or in combination with acidification. However, the consequences of elevated *p*CO_2_ levels described in this study might be worse than the potential ecological impacts described here if the investigated thermal range includes more stressful temperatures. Future studies need to go further than the relatively moderate warming scenario investigated here. Furthermore, populations naturally exposed to warmer thermal limits such as those found along the northern Chilean coast also need to be investigated in order to fully understand the combined effects of warming and acidification on this species and others with similar life histories.
